# Prevalence of pest nematodes associated with soybean (*Glycine max*) in Wisconsin from 1998 to 2021

**DOI:** 10.2478/jofnem-2023-0053

**Published:** 2024-03-30

**Authors:** A. E. MacGuidwin, D. L. Smith, S. P. Conley, K. A. Saikai

**Affiliations:** Department of Plant Pathology, 1630 Linden Drive, University of Wisconsin-Madison, Madison, WI 53706; Department of Agronomy, 1575 Linden Drive, University of Wisconsin-Madison, Madison, WI 53706; Agro-Systems Research, Wageningen University and Research, NL-6708 PB Wageningen, The Netherlands.

**Keywords:** cyst, *Heterodera glycines*, *Pratylenchus*, soybean, detection, prevalence

## Abstract

The prevalence of *Heterodera glycines* and other cyst and vermiform genera was determined from 8,009 soil samples over two decades. Prevalence of cyst nematodes for farms increased from 16% in 1998 to 1999, reaching a peak of 40%, with marked differences among Wisconsin’s nine agricultural districts in how much the odds of a positive test increased. Estimates at the sample scale also increased over time but peaked at 29%. Assay of all nematodes beginning in 2012 showed *Pratylenchus*, *Helicotylenchus*, and *Xiphinema* to be more prevalent in Wisconsin soybean fields than cyst nematodes. Prevalence estimates for *Pratylenchus* and *Helicotylenchus* for soybean and rotation crops ranged from 76 to 89% and 58 to 83%, respectively. Species identification of *Pratylenchus* from a subset of the samples revealed six species. The majority of cyst-positive samples were infested with *Pratylenchus*, and count data showed that the number of cyst eggs and juveniles per 100 cm^3^ soil was 60% lower in samples positive for *Pratylenchus*. The influence was reciprocal, as *Pratylenchus* population densities were 41% lower in samples positive for cyst nematodes, suggesting a competitive interaction. The Wisconsin soybean nematode testing program provides a useful model for estimating nematode prevalence using citizen-based surveys.

Like in other midwestern states, soybean (*Glycine max*) is an important crop in Wisconsin. More than 18,000 farms in Wisconsin harvested over 800,000 ha of soybean in 2021, with 45 of the state’s 72 counties producing over 27,218 metric tons (USDA-NASS, 2021). Soybean is most often grown in rotation with corn, and Wisconsin ranks first in the U.S. for corn silage production and ninth for corn grain (USDA-NASS, 2021). Other crops grown in rotation with soybean in Wisconsin include processing vegetables, potato, and small grains.

*Heterodera glycines* is the most important nematode pest of soybean in Wisconsin. Between 2015 and 2019, *H*. *glycines* was considered the number one pathogen affecting soybean, with over $102 million USD in total losses estimated for Wisconsin alone (Bradley et al., 2021). It is an alien species that entered the United States nearly 80 years ago and spread quickly due to its high capacity for persistence. After it was first reported in 1981 in Racine County in southeastern Wisconsin, the known distribution of *H. glycines* spread to 21 Wisconsin counties by 1997 and to 53 counties by 2019. The common name of *H. glycines*—the soybean cyst nematode (SCN)—comes from its preferred host and the ability of dead females to function as a protective casing, or cyst, for eggs. Cysts shelter the eggs until soybean plants are available, providing an escape-in-time strategy effective for years.

Other cyst-forming species of nematodes reported from Wisconsin (Phibbs et al., 2019) include *Cactodera cacti*, *C. estonica*, *C. milleri*, *C. rosae*, *C. weissii*, *H. schachtii* (Mulvey & Golden, 1983), *H. trifolii,* and *Punctodera spp*. (MacGuidwin, personal observation), but none of them reproduce on soybean. Weeds are hosts for other cyst species but are not thought to be common in Wisconsin soybean fields, as over 90% of the crop carries a biotech herbicide tolerance trait, and herbicide use is widespread (USDA-NASS, 2021).

At least ten species of nematode pests other than *H. glycines* parasitize soybean in Wisconsin and other midwestern states. The most prevalent genera reported from soybean-corn rotations in Illinois and Indiana have been *Pratylenchus spp*. and *Helicotylenchus spp*. (Ferris and Bernard, 1967; Han et al., 2022). Surveys of corn fields in Iowa, Illinois, and Ohio reported the same results (Han et al., 2021, Simon et al., 2018; Tylka et al., 2011). Some species of *Pratylenchus* are known to reduce soybean yield (Saikai and MacGuidwin, 2022; Schmitt and Barker, 1981) and a competitive symbiosis between *P. penetrans* and *H. glycines* was demonstrated in a greenhouse study using varying proportions of initial inoculum of each species (Melakeberhan and Jyotirmoy, 2003).

Estimates of the prevalence of nematode pests are used to set research priorities, inform farmers, and establish policies for transporting agricultural products. The “Widely Prevalent Plant-Parasitic Nematode List” (https://www.prevalentnematodes.org/) is a database developed by scientists and state regulatory offices to inform the public and government agencies about pests already established in each U.S. State. The list for Wisconsin includes *H. glycines* and over 40 other species. Nine species of *Pratylenchus* are listed, including *P. brachuyrus, P. hexincisus, P. pratensis, P. thornei,* and *P. vulnus,* which have not been recovered in field studies during the past thirty years (MacGuidwin, unpublished data). Conversely, a *Pratylenchus* species known to damage soybean in Illinois, *P. alleni*, is not included in the list.

Soybean farmers in Wisconsin participated in a check-off program that has funded free nematode testing since 1998. From 1998 to 2013, only assays for cyst nematodes were performed. Since 2013, each sample has been used for two assays, one for cyst nematodes and one that recovers all nematodes, including infective juveniles of cyst nematodes. Samples, collected by farmers or agricultural professionals working with farmers, are tested by professionals, who send results and information about *H. glycines* to submitters.

In this study, these data from the testing program were used to 1) determine the prevalence of cyst nematodes and test the hypothesis that the prevalence of cyst nematodes in Wisconsin has increased over time; 2) estimate the prevalence of all pest nematodes on soybean and crops rotated with soybean; 3) determine if the Wisconsin field data supports a competitive interaction between cyst and other pest nematodes; and 4) identify species of *Pratylenchus* associated with soybean in Wisconsin, with emphasis on diecious species.

## Materials and Methods

*Nematode testing program:* Sampling kits consisting of a lined bag, mailing labels, sampling instructions (https://www.thescncoalition.com/wp-content/uploads/2021/12/Scouting-and-Soil-Testing-for-SCN-Resource.pdf), and a submission form were distributed to farmers or industry representatives. The submission form requested the name, address, and contact information for the submitter and farmer, the business name of the farm, the field name and location (county and township), the current and previous crop, and the soil texture. It also asked if the field had been sampled before, and if the presence of SCN was suspected. Free testing was provided for up to four samples per farm per year. The soil was tested for nematodes by the Nematode Diagnostic Service at the University of Wisconsin-Madison from 1998 to 1999 and 2010 to 2016, and then by the commercial laboratory Pest Pros, Inc. (Plainfield, WI) from 2017 to 2021. Data records from 2000 to 2009 were lost, so they were not included in the study.

### Nematode recovery

*Cyst nematode assay:* Soil samples were tested for cyst nematodes every year using a standard cyst nematode assay. The bags of soil were placed into a large container, where clods formed by sampling utensils were broken and the soil was mixed. A 100-cm^3^ subsample of soil was measured by water displacement and placed into a bucket with additional water. The mixture was vigorously stirred and then allowed to settle for 15 seconds before it was poured over a 850-μm-mesh sieve nested over a 250-μm-mesh sieve. Debris on the 850-μm-mesh sieve was discarded and the contents of the 250-μm-mesh sieve were washed into a centrifuge tube. The sample was centrifuged twice for 4 min at 3800 rpm; the first time was to pellet the soil, and the second time was to suspend cysts in a sugar solution with a specific gravity of 1.63. The cysts were collected by passing the supernatant of the second centrifugation over a 180 μm-mesh sieve and rinsing with water to remove any remaining sugar solution. The cysts were then crushed to release eggs, following the protocol detailed by Fagihi and Ferris (2000), and counted using a stereomicroscope. Cyst-forming nematodes were not identified to species.

*All-nematode assay*: Soil samples from 2012 to 2021 were tested for all nematodes. The assay was similar to the procedure for cyst nematodes, except the nested sieves had mesh sizes of 250 μm and 38 μm. The contents of the top sieve were incubated on a Baermann funnel for 48 hrs and the contents of the bottom sieve were processed using sugar flotation and centrifugation with a sugar solution of 1.14 specific gravity. The dual assays described in detail in MacGuidwin and Bender (2012) were used for all samples processed at the University of Wisconsin-Madison and for samples processed by Pest Pros between November 1^st^ and May 31^st^.

Pest Pros incubated nematodes retained on a 325-μm-mesh sieve in Baermann funnels for 48 hours for samples received between June 1^st^ and October 31^st^.

Nematodes recovered from the Baermman funnels and centrifugation assays were observed using a stereomicroscope and counted using the following categories: cyst (*Heterodera*, *Cactodera*), *Pratylenchus*, *Xiphinema*, *Hoplolaimus*, *Longidorus*, spiral (*Helicotylenchus*, *Rotylenchus*), stunt (*Quinisulcius*, *Tylenchorhynchus*), stubby root (*Trichodorus*, *Paratrichodorus*) and ring (*Criconema*, *Criconemoides*, *Crossonema*, *Mesocriconema*, *Ogma*, *Xenocriconemoides*) nematodes. Common names were used to group genera that could not be distinguished using a stereomicroscope.

**Figure 1:**
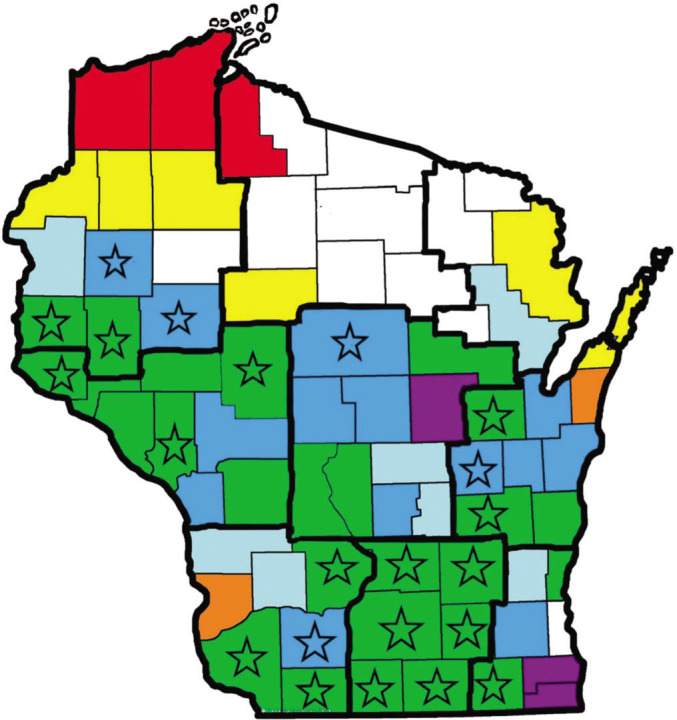
Map of Wisconsin showing counties colored according to their representation (number of bienniums) in the survey data: green = seven; dark blue = six; light blue = five; purple = four; orange = three; yellow = two; red = one; white = not included. The star symbol indicates counties with soybean planted on more than 15,000 ha in 2021. Nine agricultural districts are outlined in bold.

*Identification of Pratylenchus species: Pratylenchus* was identified to species from 100 samples. The samples were selected by geographic location to represent the full range of soybean production in Wisconsin, with 38 samples from the southern third region, 55 samples from the central third, and seven samples from the northern third of the state (Figure 1). Forty-five samples were submitted in 2019, and 55 were submitted between 2013 and 2017. Adult nematodes were processed for identification from each sample: five females from 39 male-negative samples, five males from 57 male-positive samples, and a mix of males and females from four male-positive samples. Emphasis was placed on males because five species with questionable prevalence status are diecious, with abundant males: *P. alleni*, *P. brachyurus*, *P. pratensis*, *P. thornei*, and *P. vulnus*.

Nematode DNA was extracted using the Dneasy Blood & Tissue Kit (Qiagen, Valencia, CA). If PCR was not conducted immediately, the DNA extracts were stored at −20 °C. Five micro-liters of DNA were suspended in a 25 µl reaction volume composed of 2.5 µl ddH_2_O, 2.5 µl of reverse and forward primers, and 12.5 µl of GoTaq Green Master Mix ×2 (Promega, Madison, WI). Two loci were amplified using the following primers: the forward *D2A* (5′-ACAAGTACCGTGAGGGAAAGTTG-3′) and the reverse *D3B* (5′-TCGGAAGGAACCAGCTACTA-3′), amplifying the D2–D3 region of 28S rDNA (Subbotin et al., 2006), and the forward *JB3* (5′-TTTTTT GGGCATCCTGAGGTTTAT-3′) and the reverse *JB4.5* (5′-TAAAGAAAGAACATAATGAAAATG–3′) primers for amplification of the partial COI gene of mitochondrial DNA (Derycke et al., 2010).

PCR cycling conditions for amplification of 28S rRNA were 94 ºC for 5 min, followed by 40 cycles of denaturation at 94 ºC for 30 sec, annealing at 55 ºC for 30 sec, and extension at 72 ºC for 1 min, with the final step at 72 ºC for 10 min. PCR cycling conditions for COI were: 94 ºC for 5 min, 5 cycles of denaturation at 94 ºC for 30 sec, annealing at 54 ºC for 30 sec with temperature decreasing by 1 ºC for each cycle, and extension at 72 ºC for 30 sec followed by 35 cycles of 94 ºC for 30 sec, 50 ºC for 30 sec, and 72 ºC for 30 sec, and a final extension of 10 min at 72 ºC. Five microliters of the PCR products were resolved by electrophoresis on 1.0% agarose gel mixed with 2.5 µl of SYBR Safe DNA Stain (Invitrogen, Waltham, WA) at 70V for 1 hour. The presence of DNA in the PCR products was confirmed by Gel Logic 200 Imaging System (Kodak Alaris, Rochester, NY). The crude PCR products were sent to Functional Biosciences (Madison, WI) for purification and Sanger-sequencing.

Approximately 700 bp and 440 bp of nucleotide sequences were read without gaps by the 18S, 28S and COI primers in this study. The DNA sequences were edited with Finch TV (Geospiza Inc, Seattle, WA), and aligned with MUSCLE in MEGA7 (Kumar et al., 2016) using default parameters, with the gap opening penalty set at 15 and the gap extension penalty at 6.66. Alignments were further adjusted by inspection. A complemented sequence was constructed by overlapping sequence reads with forward and reverse primers. Basic Local Alignment Search Tool (BLAST) from the National Center for Biotechnology Information (NCBI) was used to compare our complemented sequences to the GenBank sequence database.

The previously published species-specific primer for *P. penetrans*, *PPEN* (5′-TAAAGAATCCGCAAGG ATAC-3′), based on the D3 region of the 28S rDNA (Al-Banna et al. 2004), was tested for its ability to distinguish *P. penetrans* from other sexual species putatively present in Wisconsin. PCR was performed on 17 culture isolates using the species-specific primer with the reverse primer, D3B, following the protocol used for the 28S rDNA. DNA amplification was confirmed by gel electrophoresis as described. Species were assigned to samples if at least one individual was a > 99% match to a species’ D2–D3 and CO1 reference sequences published in GenBank, and in the case of *P. penetrans*, was positive for the PPEN primer.

*Data analysis:* Data sets consist of 1) all samples submitted to the program, 2) samples filtered by information provided on the submission form such as crop sampled or cropping history, and 3) farms where the samples were collected. Names of the businesses and farmers, as well as their addresses, were cross referenced so that each farm was represented only for the first submission. If multiple samples were received from the farm, the maximum count for each nematode was recorded. Geographical analyses were based on the nine agricultural districts used by USDA’s National Agricultural Statistics Service to represent Wisconsin’s 72 counties: southwest, south central, southeast, west central, central, east central, northeast, north central, and northeast districts (Figure 1). Data were collated to represent seven biennia for temporal analyses: 1998 to 1999, 2010 to 2011, 2012 to 2013, 2014 to 2015, 2016 to 2017, 2018 to 2019, and 2020 to 2021.

Prevalence was calculated as the percentage of samples or farms positive for cyst nematodes from 1998 to 2021 or all nematodes from 2012 to 2021. Data analyses were performed using SAS/STAT software version 9.4 (SAS Institute, Inc, Cary, NC, USA). Percentages were analyzed as *n*-way frequency tables using the SAS PROC FREQ Procedure and compared using Fisher’s Exact Test. The chi square option and Fisher’s Exact Test were used for 2 × 2 comparisons. A right-sided probability value (p) was used to test the null hypothesis that cyst nematode prevalence did not increase over time. A two-sided *p* value was used to test the null hypotheses that prevalence was the same for all agricultural districts, crops, or pest nematode groups. McNemar’s Test was used to compare prevalence of cyst nematodes from the same farm for the first and last biennium.

Logistic regression using the PROC LOGISTIC procedure was used to generate odds ratios to describe the association between a positive test result for cyst nematodes and other factors. Multiple models for cyst-positive farms were used to study changes in cyst prevalence over time. In one model with only main effects of biennium and district, the time variable was parameterized using the “reference” option with the 1998–1999 biennium as the reference. In another model with main effects, time was parameterized using the “ordinal” option so that each biennium was compared to the one before. Odds ratios for each combination of biennium and agricultural district were generated in a third model using the “contrast” option. The PROC LOGISTIC procedure was also used to calculate the odds of samples being positive for other pest nematodes versus cyst nematodes. Odds ratios were only reported if the Wald statistic for the log odds estimate was significant (*p* < 0.05).

The nonparametric Kruskal-Wallis Test, generated using the PROC NPAR1WAY procedure, was used to examine the interaction of cyst nematodes with *Pratylenchus spp*. Counts (100 cm^3^ soil) of a genus in the presence and absence of the other nematodes were compared. Lower population densities in samples containing both genera, as compared to samples containing only one, were considered to indicate competition symbiosis.

## Results

The testing program received 8009 samples collected from more than 2000 farms in 61 counties from 1998 to 1999 and 2010 to 2021 (Table 1). Nineteen percent of the samples were submitted before June 30^th^, 39% between July 1 and September 30^th^, and 42% after October 1. Most samples (95.7%) came from fields where soybean was alternated with a nonhost crop for SCN for one (82.4%) or two (13.3%) years. Corn was the most common nonhost crop in the rotation, with cereal grain a distant second, followed by specialty and forage crops. Soil texture ranged from sand to silty clay, with sandy loam-to-silt-loam texture classes the most prevalent. The response rate was very low for soil texture, so no analyses using this information were performed.

**Table 1.** Program information and prevalence of cyst nematodes for all samples and every farm that was assayed for cyst nematodes.

**Biennium**	**Number of counties sampled**	**Number of samples**	**Number of farms sampled^a^**	**% samples positive for cyst nematodes**	**% farms positive for cyst nematodes^b^**	**Odds Ratio current biennium vs 1998–1999**
1998 – 1999	42	659	427	16	16	
2010 – 2011	38	379	150	22^*^	23^*^	1.84^*^
2012 – 2013	51	1121	377	22	31^*^(*p* = 0.06)	2.59^***^
2014 – 2015	54	1268	413	22	30	2.63^***^
2016 – 2017	49	1132	299	26^*^	40^**^	3.87^***^
2018 – 2019	49	1588	334	29^*^	43	4.86^***^
2020 – 2021	51	1862	320	27	39	3.75^***^

a each farm is represented only once and for the first time samples were submitted

b increase in prevalence from the preceding biennium according to Fisher’s Exact Test

* *p* < 0.05;

** *p* < 0.01;

*** *p* < 0.0001.

*Prevalence of Cyst Nematodes 1998 to 2021:* The prevalence of cyst-positive farms in the baseline biennium was 16% and increased in the 2010-to-2011 biennium and again in the 2012-to-2013 and 2016-to-2017 biennia (Table 1). As compared to the baseline biennium, the odds of a farm in Wisconsin being cyst-positive increased by 84% after 13 years and 287% after 19 years. Seventy-four percent of the cyst-positive farms first sampled from 2018 to 2021 were infested with fewer than 2,000 cyst eggs and juveniles per 100 cm^3^ soil. Comparison of the same 51 farms over time also showed an increase (*p* = 0.0001) from 18% cyst-positive (1998–1999) to 82% cyst-positive (2018–2021). Eighty-eight percent of those cyst-positive farms were infested with fewer than 2,000 cyst eggs and juveniles per 100 cm^3^ soil on the most recent sampling date. Prevalence estimates based on samples increased over time, but peak values were lower (*p* < 0.0001) than estimates based on farms.

There was a significant (*p* < 0.0001) biennium × district interaction in the logistic model for cyst-positive farms, suggesting that the location of farms within the state impacted cyst status. The proportion of cyst-positive farms in the baseline biennium ranged from < 10% in the southwest, central, and east central districts to 27–29% in the west central, northwest, and southeast districts. At the district level, the proportion of cyst-positive farms first increased in the 2010–2011 biennium for the southwest (*p* = 0.02), south central (*p* < 0.01), and central (*p* = 0.02) districts, during the 2012-to-2013 biennium for the east central (*p* < 0.0001) and southeast (*p* = 0.10) districts, during the 2018-to-2019 biennium for the west central district (*p* = 0.01), and never for the northwest district. In the 2020-to-2021 biennium, the odds of a farm being cyst-positive in the west central, south central, east central, southeast, and central districts were 2.3, 2.5, 7.1, 17.1, and 18.6 times higher (*p* < 0.04) than in the baseline biennium, respectively. Estimates of the log odds for the northwest and southwest districts were not significant.

*Prevalence of All Pest Nematodes 2012 to 2021:* At least one pest nematode was recovered from over 97% of the 6971 samples submitted and 98% of the 1743 farms tested from 2012 to 2021. Only the cyst-forming genera showed any temporal patterns of prevalence, so time was not included in analyses. Genus assignment was not confirmed by molecular diagnostics, but the majority of specimens assigned to the spiral and stubby root category appeared to be *Helicotylenchus* and *Paratrichodorus spp.,* respectively. Prevalence of genera for the stunt category was not possible to estimate, but it appeared that *Tylenchorrhynchus* and *Quinisculcius* were common. Cysts were not examined for many of the samples, so the frequency of genera other than *Heterodera* could not be estimated. Genera recovered in fewer than 0.5% of the samples included *Meloidogyne* and *Ditylenchus*. The genus *Paratylenchus* was present but not enumerated by the commercial lab.

At both the sample and the farm level, *Pratylenchus* was the most prevalent genus detected and *Longidorus* the least. (Table 2). There were significant differences in prevalence among the pest nematode categories. Pairwise comparisons showed the prevalence of all other nematodes to be different (*p* < 0.0001) from that of cyst nematodes. The odds of a farm testing positive were higher for *Pratylenchus*, *Xiphinema*, and spiral nematodes than cyst nematodes. The odds of a farm receiving a positive test result for *Longidorus spp*., *Hoplolaimus spp*., stubby root nematodes, or ring nematodes was over 83% lower than for cyst nematodes.

**Table 2.** Prevalence of all pest nematode from 2012 to 2021 at the sample and farm scale.

**Pest nematodes^a^**	**samples (6971)**	**farms (n = 1743)**	**Odds ratio^c^**

	**% positive**	**% positive**	**(*p* < 0.0001)**
*Pratylenchus spp*.	84	92	21.27
Spiral nematodes	70	78	6.33
*Xiphinema spp*.	33	46	1.52
Cyst nematodes	26	36	reference
Stunt nematodes	18	28	0.68
Stubby Root nematodes	6	9	0.17
*Hoplolaimus spp*.	5	7	0.13
Ring nematodes	3	6	0.12
*Longidorus spp*.	1	2	0.03
Chi Square statistic^b^	6,805	26,257	
*P*	< 0.0001	< 0.0001	

a spiral = *Helicotylenchu*s and *Rotylenchus spp.;* cyst = *Heterodera glycines* and *Cactodera*, *Heterodera*, and *Punctodera spp*.,; stunt = *Tylenchorrhynchus* and *Quinisculcius*; ring = *Criconema, Criconemoides*, *Mesoscriconema*, and *Ogma spp.*

b Chi Square statistic for the null test of equal prevalence for all nematodes

c Odds ratio of farms testing positive for a particular nematode versus cyst nematodes

The majority of the samples were collected from soybean and corn, so prevalence estimates were also examined at the crop level (Table 3). Consistent with the general analysis, *Pratylenchus spp*. and spiral nematodes ranked first and second, respectively, and ring and *Longidorus spp*. ranked lowest for all crops. *Xiphinema spp*., *Hoplolaimus spp*., and stunt nematodes had the greatest prevalence estimates for a crop other than soybean. Prevalence estimates of cyst nematodes were similar for samples collected in soybean, wheat, and vegetable fields, but were lower in corn and alfalfa samples were than in soybean.

**Table 3.** Prevalence of pest nematodes in samples shown by the crop sampled.

**crop**	**n**	***Pratylenchus***	**Spiral**	***Xiphinema***	***Hoplolaimus***	**stunt**	**stubby-root**	**ring**	***Longidorus***	**cyst**
soybean	3309	84	73	31	3	16	5	2	0	29
corn	2188	84	67^*^	37^*^	6^*^	18^*^	5	2	1	20^*^
wheat	75	89	83	23	5	31^*^	5	3	1	27
alfalfa	32	81	69	41	3	44^*^	0	6	0	3^*^
vegetables	38	76	58	8	0	37^*^	8	3	0	21
Chi Square		4	27	37	31	45	3	3	15	64
*p*		0.48	<0.0001	<0.0001	0.0001	<0.0001	0.64	0.53	0.03	<0.0001

* Difference (*p* < 0.001) in prevalence on a particular crop as compared to soybean according to Fisher’s Exact Test is shown for each pest nematode and the Chi Square statistic for the null hypothesis of equal prevalence among crops.

The majority of cyst-positive samples were infested with *Pratylenchus spp*., so sample count data was used to evaluate the influence of cohabitation on nematode population densities (Table 4). The number of cyst eggs and juveniles per 100 cm^3^ soil was 60% lower in samples positive for *Pratylenchus spp*. The influence was reciprocal as *Pratylenchus spp*. population densities were 41% lower in samples positive for cyst nematodes. The mutual suppression of population density satisfied the criterion suggesting competition between the two pest taxa.

**Table 4.** Population densities of *Pratylenchus spp.* or spiral nematodes and cyst-forming species in the presence (+) and absence (−) of each other in samples collected from soybean fields in Wisconsin from 2012 to 2021.

**Comparison**	**Samples**	**Nematodes per 100 cm^3^ soil^a^**	**Kruskal Wallis**	

	**n**	**Mean (Maximum)**	**Chi Square**	***p***
*Pratylenchus* / − Cyst	2349	121 (4960)		
*Pratylenchus* / + Cyst	960	71 (1508)	90.63	< 0.0001
Cyst / − *Pratylenchus*	518	1144 (44364)		
Cyst / + *Pratylenchus*	2791	452 (49494)	30.41	< 0.0001

a Mean population densities of the first nematode listed are shown.

*Species of Pratylenchus*: Three parthenogenetic *Pratylenchus* species, *P. crenatus*, *P. neglectus*, and *P. scribneri*, were identified from 39 male-negative samples (Table 5). All three species were detected in fields spread across the state planted with soybean (*P. crenatus* and *P. neglectus*), wheat (*P. scriberi* and *P. neglectus*), and corn (all three species). Multiple species were recovered from 31% of the samples, including three samples positive for a dioecious species.

**Table 5.** *Pratylenchus* species identified from 100 soil samples submitted to a nematode testing program in Wisconsin.

**Species Assignment**	**Male-Positive Samples (n = 61)**		**Male - Negative Samples (n = 39)**	

	**No. of Samples**	**No. of Counties**	**No. of Samples**	**No. of Counties**
*Pratylenchus fallax*	3	3	0	0
*Pratylenchus dakotaensis*	3	3	1	1
*Pratylenchus alleni*	7	7	0	0
*Pratylenchus penetrans*	49	35	2	2
*Pratylenchus scribneri*	0	0	5	5
*Pratylenchus crenatus*	1	1	19	18

Four *Pratylenchus* species, *P. alleni, P. dakotaensis*, *P. fallax*, and *P. penetrans,* were identified from the 61 male-positive samples (Table 5). One sample was positive for *P. alleni* and *P. penetrans*, with the remainder of the samples positive for only a single species. *P. penetrans* was widely prevalent throughout the state in samples from soybean, corn, kidney bean, and wheat. *Pratylenchus dakotaensis* and *P. alleni* were detected from corn and soybean samples in the southern and central regions of the state and *P. fallax* were detected from corn samples across the central region.

## Discussion

The prevalence of cyst nematodes increased in Wisconsin from farms participating in a nematode testing program for over two decades. This increase, demonstrated at both the farm and sample level of resolution, confirms hypotheses of an increased population of nematodes that was based on the number of infested counties (Tylka and Marett, 2021). It is interesting that our prevalence estimates peaked at about 40% in the 2020 to 2021 biennium, at a level well below the estimated prevalence of cyst nematodes in neighboring states of Illinois and Minnesota from 1995 to 1996 (Workneh et al., 1999). Estimates for cyst nematode prevalence in Illinois from 2018 to 2020 were twofold greater than our estimates for 2018 to 2021 in Wisconsin, but the percentage of cyst-infested farms exceeding 2,000 nematodes per 100 cm^3^ soil were similar for the two states (Kleczewski et al., 2023).

We did not identify cyst nematodes to species, but it is almost certain that the majority of specimens categorized as cyst nematodes were the soybean cyst nematode, *Heterodera glycines*. We have multiple lines of evidence to support this conclusion. The morphology of cysts we examined was consistent with other studies descriptions of *H. glycines* and cyst nematodes from 244 farms reproduced on soybean and were characterized for Hg Type (MacGuidwin, unpublished).

*Heterodera trifolii* and *H. schachtii* were reported for Wisconsin, but the crops able to support the reproduction of these species represented less than 1% of the samples with the crop identified. The primary hosts of *Cactodera* species are weeds, and herbicides are used on more than 90% of the corn and soybean acreage in Wisconsin, which accounted for most of the samples submitted to the testing program.

More farms in Wisconsin were infested with *Pratylenchus spp*., *Xiphinema spp*., and spiral nematodes than with cyst nematodes. These pests have very wide host ranges, and their prevalence did not vary much among the field crops represented in our survey. Our prevalence estimates of *Pratylenchus spp*. are similar to reports from corn surveys, and our estimates of population density in soybean samples greatly exceeded those reported from corn (Han et al., 2018; Simon et al., 2021). Given the ubiquity and apparent reproductive success of *Pratylenchus* spp. in soybean fields, it is misleading to refer to this pest as a “corn nematode” in the manner that it often appears in marketing campaigns for commercial products.

Our data showing lower population densities of cyst and *Pratylenchus spp*. when they occur together is consistent with a greenhouse study comparing single versus co-inoculation of *H. glycines* and *P. penetrans* (Melakeberhan, and Jyotirmoy, 2003). The greenhouse study had an 18-day duration and focused on early events during the life cycle of a single cohort of nematodes, while our results reflect reproductive success smoothed over many cohorts and points in the life cycle. Even though data from the two studies are not directly comparable, the similar outcomes suggest a competitive interaction of cyst and *Pratylenchus spp*. that warrants further study.

This study suggested that diagnosing *Pratylenchus* at the genus level belies the diversity present and misses opportunities for economical nematode management. For example, empirical models relating nematode population densities of *P. penetrans* to soybean (Saikai and MacGuidwin, 2022) and corn yield (MacGuidwin and Bender, 2012) are likely to overestimate the threat of *P. crenatus*, a less-pathogenic species (Willis et al., 1982) that is present throughout Wisconsin. Adding a small-grain cereal to the rotation may dampen the increase of nematodes in a small number of fields infested with *P. scribneri*, but fuel nematode population densities in many fields infested with the highly prevalent *P. neglectus*, according to their published host ranges (nemaplex.ucdavis.edu). The majority of our samples as well as other surveys (Mkete et al., 2011; Ozbayrak, 2019) contained only one species of *Pratylenchus*, so species-specific management is feasible. Even a putative assessment based on the presence of males would be helpful for farmers in Wisconsin, as 97% of the male-positive samples contained either *P. penetrans*, *P*.*alleni*, or *P. dakotaensis,* which are all known to be highly damaging to soybean (Acosta, 1982; Chowdhury and Yan, 2021; Saikai and MacGuidwin, 2022).

Species identifications from this study suggest additions to, and deletions from, the list of *Pratylenchus* species considered widely prevalent in Wisconsin (https://www.prevalentnematodes.org/). *P. alleni*, *P. dakotaensis*, and *P. fallax* should also be added due to by the number of counties with infested fields that are at least 125 miles apart. *P. alleni* has been reported in two bordering states, but Wisconsin is unique or among only a few states reporting *P. fallax* and *P. dakotaensis*. Zero detection of *P. brachyurus* and *P. hexincisus* is a good reason to remove these species from the list, because both have a high rate of reproduction on corn and soybean (Rodrigues et al., 2014; Zirakparvar, 1980) and are highly prevalent *Pratylenchus* species on these crops where they do occur (Machado, 2014; Norton, 1989). Failure to detect three dioecious species, *P. pratensis*, *P. thorneii,* and *P. vulnus*, given our focus on adult males, is strong evidence they are not widely prevalent in Wisconsin, but this needs to be confirmed by sampling the fruit and vegetable crops that account for many records of their occurrence.

Prevalence estimates of nematodes are important for alerting farmers about the risk pest nematodes impose to their crops. Prevalence can be measured at any scale and time frame, so the relevance of prevalence estimates is goal-dependent. Surveys at a point in time are the most commonly used method of estimating the status of *H. glycines* or other pest nematodes in soybean-corn production systems (Han et al., 2022; Kleczewski et al., 2023; Workneh et al., 1999). The value of collecting data over a wider time frame was illustrated by our study, as not all agricultural districts saw an increase in cyst nematodes as compared to the 1997–1998 biennium at the same time. Soybean acreage did not seem to be a strong factor for the spread of cyst nematodes, as prevalence increased in the 2010–2011 biennium for both the south central district, which had a high level of soybean acreage, and the central district, which had a low level of soybean acreage.

The Wisconsin soybean nematode testing program provides a useful model for estimating nematode prevalence using citizen-based surveys. The program was an efficient way to collect a lot of nematode samples over a long time period for both immediate and retrospective use. Bias of volunteer participants is a potential problem, but expansion of the program to include all pest nematodes reduced bias for a particular nematode pest. This was evidenced by the timing of sample collection, the diversity of rotation crops sampled, and the finding that 75% of cyst-positive samples had fewer than 2000 eggs per 100 cm^3^ of soil and were probably asymptomatic. Adding the total nematode assay to the program also revealed the potential risk from *Pratylenchus spp*. and improved detection of *H. glycines* when population densities were very low. Lessons learned include the value of using farm addresses to link present-day conditions with historical data, and the need to incentivize submitters to provide ancillary information for each sample.
